# Micro-Organisms and the Sweat

**Published:** 1892-06

**Authors:** 


### MICRO-ORGANISMS AND THE SWEAT.

Medical science sometimes makes a real advance when it undergoes a revolution. It is only during a very recent period that it has had to modify its knowledge and views concerning the mechanism and role of fever in the economy, and instead of regarding it as a purely pathological process against which we should concentrate our efforts, it is now recognized and conceded to be, within certain limits, a physiological and conservative process, by which the organism protects and restores a lost equilibrium. In the symptom fever we have an index to guide us in the changes incident to leucocytosis and its concomitants of chemotaxis and phagocytosis which are the vital activites of the cell and constitute the veritable *vis medicatrix naturæ.* It is in these important functions of the cells that we find the phenomena by which disease is prevented, arrested and cured, and the degree of perfection to which they are operative and effective is in a large measure directly proportional to the presence of fever. In this instance we have an illustration of a complete revolution in the conceptions and treatment of disease. Again, in the office or function of the skin we have radical views to change for the older ones. Brunner has recently shown from a number of experiments that the microbes of a number of diseases are eliminated by the sweat, and that, indeed, in a number of diseases this is the chief means by which they are thrown off from the organism. After thorough sterilization of the skin of animals, Brunner injected a culture of staphylococcus aureus into a hog, one of anthrax into a cat, and one of micrococcus prodigiosus into a sucking pig. In all three cases the microbes injected were found in the sweat and the saliva.

This is one of the most important discoveries, from both practical and theoretical points of view. It gives to crises, accompanied by profuse perspirations which may be produced artificially or naturally, a still more important place than they formerly held. It shows that there is great danger of the patient reabsorbing the secretions. Again in discharging abscesses, erysipelas or other infectious diseases, there is great danger to the patient and his attendants from these sources. It would be a good plan with these facts before us, to subject all linen from a patient suffering from these disorders, after each profuse perspiration, to a change or disinfection in order to lessen the liability to reinfection or new infection from them as sources.

In fear, therefore, the profuse sweat is an attempt of the organisms to rid itself of the morbific agents. It should be encouraged and the products rendered inocuous. J. W. B.

---

Dr. J. M. Keating, of Colorado Springs, has resigned the editorship of the *Climatologist.*

---

Dr. William Cobb Whitfield, of La Grange, N. C., has been elected superintendent of the new Orphan Home, established in Goldsboro, by the Odd Fellows of North Carolina.

---

The American Neurological Association will hold its annual meeting in New York, at the Acadamy of Medicine, on June 22d, 23d and 24th, under the presidency of Dr. C. L. Dana.

---

EXAMINATION QUESTIONS.—Among the many recent developments of our friends, the belly-rippers, may be mentioned the two following interrogatories to be propounded in future green-room examinations:

"Give the diagnosis of abdominal affections."

"If the patient is a male, he has appendicitis; if a female, pyosalpinx."

"What is the indication for abdominal section?"

"The ability to pay a satisfactory fee."—Ex.

